# Microbial Conversion of Vegetable Oil to Hydroxy Fatty Acid and Its Application to Bio-Based Polyurethane Synthesis

**DOI:** 10.3390/polym10080927

**Published:** 2018-08-19

**Authors:** Tuan Kiet Tran, Prasun Kumar, Hak-Ryul Kim, Ching T. Hou, Beom Soo Kim

**Affiliations:** 1 Department of Chemical Engineering, Chungbuk National University, Cheongju 28644, Korea; ttkiet1987@gmail.com (T.K.T.); prasun@cbnu.ac.kr (P.K.); 2 School of Food Science and Biotechnology, Kyungpook National University, Daegu 41566, Korea; hakrkim@knu.ac.kr; 3 Renewable Product Technology Research Unit, National Center for Agricultural Utilization Research, ARS, USDA, Peoria, IL 61604, USA; ching.hou@ars.usda.gov

**Keywords:** 7,10-dihydroxy-8(E)-octadecenoic acid, olive oil, polyol, polyurethane, *Pseudomonas aeruginosa* PR3

## Abstract

New polyurethanes were synthesized based on dihydroxy fatty acid obtained by the microbial conversion of olive oil. Monounsaturated 7,10-dihydroxy-8(E)-octadecenoic acid (DOD) was produced from olive oil by *Pseudomonas aeruginosa* PR3 and reacted with hexamethylene diisocyanate (HMDI) at different ratios to form polyurethanes. Fourier transform infrared spectroscopy and gas chromatography/mass spectrometry confirmed the synthesis of DOD. The thermal and tensile properties of the polyurethanes were investigated by differential scanning calorimetry, thermogravimetric analysis, and a universal testing machine. At an isocyanate/hydroxyl ratio of 1.4, the polyurethane exhibited an elongation at break of 59.2% and a high tensile strength of 37.9 MPa. DOD was also mixed with polycaprolactone diol or polyethylene glycol at different weight ratios and then reacted with HMDI to produce new polyurethanes of various properties. These polyurethanes displayed higher elongation at break and good thermal stability. This is the first report on the synthesis of polyurethanes based on DOD produced by the microbial conversion of vegetable oil.

## 1. Introduction

Polyurethanes (PUs) are flexible polymeric materials containing a urethane group in their structure [1]. Due to their unique mechanical, physical, biological, and chemical properties, PUs are one of the most important polymers in many potential applications. They attracted researchers’ attention owing to their cost-effective synthesis and use in a variety of commercial applications. PUs have been widely used in a variety of fields such as medicine, construction engineering, coatings, sealants, adhesives, foams, and composites because they can be shaped into the form of fibers, films, castables, thermoplastics, and foams [2,3,4,5,6]. PUs are typically synthesized by reacting a polyol containing two or more hydroxyl groups (OH) with an isocyanate containing two or more isocyanate groups (NCO). There are a limited number of isocyanates that can be used in the chemical industry due to their toxicity. In contrast, many polyols are available, but most of the materials used to synthesize polyols are petroleum-based and thus cause environmental problems [7]. Therefore, the production of isocyanates and polyols using renewable resources has received considerable interest from many scientists around the world [8,9,10,11,12,13].

In recent years, renewable resources for raw materials have not only contributed to sustainable development but also helped to address environmental problems, waste disposal, and non-renewable resource depletion. In this context, vegetable oils are the most ideal renewable resources and have increasingly been utilized in the spotlight of the chemical and polymer industries. These oils possess certain reliable characteristics including universal convenience, inherent biodegradability, low cost, and low eco-toxicity [14]. The main sources of oil, namely palm trees, soybeans, rapeseed, cotton, sunflower, palm kernel, olive, and coconut, are being explored for beneficial improvements in research and development. As a result, bio-oil derived polymers or composites are widely applied to paints, coatings, adhesives, and biomedicine. In particular, vegetable oils are triglycerides containing long chain fatty acids such as oleic acid, linoleic acid, and linolenic acid that can be used to produce hydroxy fatty acids (HFAs). HFAs are known to have higher viscosity and reactivity than other fatty acids. Due to these features, HFAs have enormous potential in industrial applications including plastics, waxes, nylons, lubricants, cosmetics, and paint additives. Since HFAs were originally found in limited amounts in the plant system, recent studies have focused on microbial conversion processes of various unsaturated fatty acids to HFAs. *Pseudomonas aeruginosa* PR3 is a known strain with the ability to convert many unsaturated fatty acids to mono-, di-, and tri-hydroxy fatty acids [15,16,17,18,19,20]. Among fatty acid substrates, oleic acid was efficiently used by strain PR3 to produce 7,10-dihydroxy-8(E)-octadecenoic acid (DOD) [21,22]. Due to the high content of oleic acid, olive oil can be used as a preferred substrate for DOD production by the PR3 strain [23,24]. DOD is a novel compound with two hydroxyl groups that can react completely with isocyanates to form PUs.

This study presents the first proof-of-concept for PU synthesis using DOD produced by *P. aeruginosa* PR3, using olive oil as a substrate. Here, the preparation of PU was carried out through a reaction mediated by hexamethylene diisocyanate (HMDI) in different ratios. In addition, DOD was mixed with different weight ratios of polyethylene glycol (PEG) (molecular weights 200, 2000, and 20,000) or polycaprolactone diol (PCLDO) (molecular weight 2000) and reacted with HMDI to form new PUs. Fourier transform infrared spectroscopy (FTIR) and gas chromatography/mass spectrometry (GC/MS) were used to verify the structure of the DOD. The prepared PUs were characterized by FTIR, differential scanning calorimetry (DSC), and thermogravimetric analysis (TGA). The tensile properties of the PUs were also measured and compared using a universal testing machine (UTM).

## 2. Materials and Methods

### 2.1. Materials

Olive oil (containing 73% oleic acid) was obtained from Samchun (Seoul, Korea). PCLDO (molecular weight 2000), PEG (molecular weights 200 and 2000), and dibutyltin dilaurate (DBTDL) were purchased from Sigma-Aldrich (St. Louis, MO, USA). PEG (molecular weight 20,000) was purchased from Fluka (Buchs, Switzerland). HMDI was purchased from Daejung (Siheung, Korea). All other reagents were of analytical grade.

### 2.2. Media and Cultivation Conditions

*P. aeruginosa* PR3 (NRRL strain B-18602) was used in this study. The standard growth medium consisted of 4 g/L glucose, 1 g/L yeast extract, 4 g/L K_2_HPO_4_, 1 g/L (NH_4_)_2_HPO_4_, 0.1 g/L MgSO_4_, 0.056 g/L FeSO_4_, and 0.01 g/L MnSO_4_. The medium was adjusted to pH 8.0 by dilute phosphoric acid [19]. The strain was grown aerobically in a 500-mL flask containing 100 mL of the standard medium at 28 °C with shaking at 200 rpm.

### 2.3. Production of DOD from Olive Oil

After 24 h of cultivation on glucose, 1.1 mL (1.0% *v*/*v*) olive oil was added to the medium for DOD production and the mixture was incubated for an additional 72 h. After the cultivation period (total 96 h), the medium was acidified to pH 2 with 6N HCl, then extracted twice with an equivolume mixture of ethyl acetate and diethyl ether [23]. Finally, the solvents were removed by a rotatory evaporator and the product was washed with n-hexane and recrystallized in ethyl acetate. DOD was filtered and dried using a solvent dryer. All experiments were conducted independently and repeated three times. Data are represented as the mean ± standard deviation.

### 2.4. Preparation of PUs Based on DOD

One gram of DOD was mixed with 1% *w*/*v* DBTDL and 3 mL chloroform in a beaker for 5 min at room temperature. HMDI was added to the beaker and mixed further for 10 min. The molar ratio of NCO to OH was set at 1.0, 1.2, 1.4, and 1.6 by varying DOD and HMDI contents. Then, the mixture was poured onto a glass plate and post-cured at 80 °C in a vacuum oven for 2 h [12]. The solvent was evaporated and the polymer films obtained after post-curing was peeled off from the plate. Films sliced into specific dimensions were used for thermo-mechanical testing.

### 2.5. Preparation of PUs Based on DOD and PEG or PCLDO

One gram of polyol was mixed with 1% *w*/*v* DBTDL and 3 mL chloroform in a beaker for 5 min at room temperature. As a polyol, DOD and PEG (molecular weights 200, 2000, and 20,000) or PCLDO (molecular weight 2000) were mixed at various weight ratios. HMDI was added to the beaker at a final molar NCO/OH ratio of 1.4 and mixed further for 10 min. The mixture was casted on a glass plate and heated to 80 °C in a vacuum oven for 2 h.

### 2.6. Analytical Methods

#### 2.6.1. GC/MS

For GC analysis, the sample was esterified first by diazomethane for 10 min at room temperature and then derivatized with the mixture of trimethylsilyl imidazole (TMSI) and pyridine (1:4, *v*/*v*) for 45 min. The derivatized samples were analyzed by GC (Agilent model 6890N, Agilent, Santa Clara, CA, USA) equipped with an HP-5 column (30 m × 320 μm × 0.25 μm). The injection volume was 1 μL. The column was initially kept at 70 °C and raised to 200 °C at 20 °C/min. After holding for 1 min at 200 °C, it was further increased to 240 °C at 0.7 °C/min followed by holding at 240 °C for 15 min [19]. The detector and injector temperatures were 280 °C and 230 °C, respectively. The methyl ester of heptadecanoic acid was used as an internal standard for quantification. The product structure was confirmed using GC/MS analysis (Agilent 6890/5973i GC/MSD, Agilent, Santa Clara, CA, USA) with a temperature gradient as follows: 70 °C to 170 °C at 20 °C/min, holding at 170 °C for 1 min, 170 °C to 250 °C at 5 °C/min, and holding at 250 °C for 15 min. The helium flow rate was 0.67 mL/min. For MS setup, ionization energy was maintained at 70 eV. The injection volume of each sample was 1 μL. The peaks were identified by comparing their fragmentation patterns with those of reference compounds.

#### 2.6.2. FTIR

FTIR analysis was performed on a Nicolet model Magna-IR 200 FTIR spectrometer (Thermo Fisher Scientific, Waltham, MA, USA). The thin films of DOD and all PU samples were made using potassium bromide (KBr) pellets with a sample/KBr ratio of 1:100. Absorbance spectra were collected at wave numbers in the range of 4000–400 cm^−1^ as described previously [25].

#### 2.6.3. DSC and TGA

DSC thermograms were obtained by using DSC Q2000 (TA Instruments, New Castle, DE, USA). Each 10-mg sample was cured dynamically by three steps. First, the samples were heated from 30 °C to 200 °C at 20 °C/min and maintained at 200 °C for 5 min. They were cooled to −40 °C at −20 °C/min and maintained at −40 °C for 5 min. Finally, they were heated again to 200 °C at 10 °C/min. TGA experiments were conducted by Discovery TGA (TA Instruments, New Castle, DE, USA) to determine the thermal decomposition behavior of PUs. All samples were heated from room temperature to 800 °C at a heating rate of 10 °C/min under N_2_ gas flow (60 mL/min).

#### 2.6.4. Tensile Properties

To measure the tensile properties of the PUs, polymer samples were prepared in dimensions of 50 mm × 10 mm. The test was performed using UTM (LR-30 K, Lloyd Instruments, Hampshire, UK) with a load cell of 1 kN and a crosshead speed of 5 mm/min at room temperature according to ASTM D638 [26,27]. The experiments were replicated at least five times.

## 3. Results and Discussion

The natural presence of at least one unsaturated fatty acid in the triacylglyceride structure of vegetable oils makes them an advantageous alternative to petroleum-based polyols. Incidentally, *P. aeruginosa* PR3 can convert these unsaturated fatty acids to HFAs through the action of triolein-induced lipase [19]. The resulting bio-based polyols may function as well as synthetic polyols for the generation of PUs using an isocyanate catalyzed reaction. Therefore, the aim of the present study was a proof-of-concept, i.e., to evaluate the efficacy of DOD for new bio-based PU synthesis.

### 3.1. Production of DOD from Olive Oil

**Figure 1:**
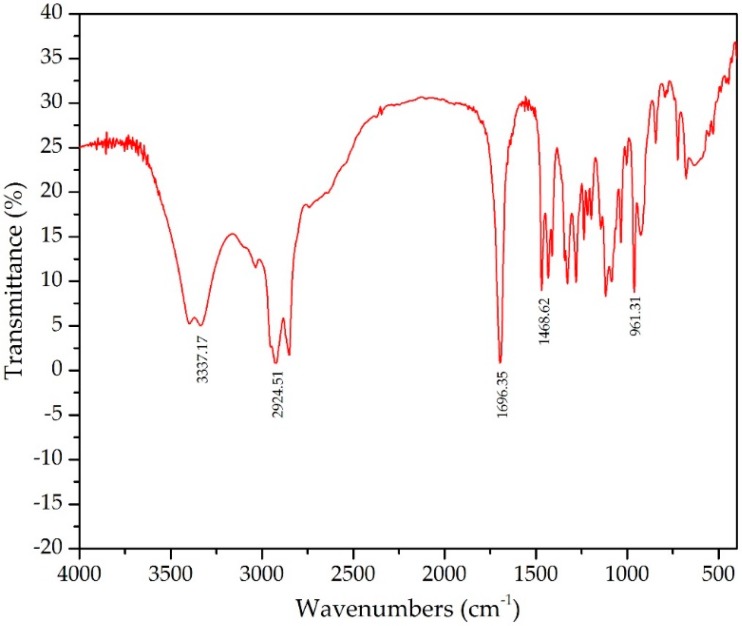
FTIR spectra of the product produced from olive oil by *Pseudomonas aeruginosa* PR3.

Olive oil was used in this work as a renewable and inexpensive model substrate for HFA production. *P. aeruginosa* PR3 grown on glucose for 24 h could effectively convert 1% olive oil to DOD after an additional 72 h of cultivation. The cultures were treated with organic solvents to extract the product and subsequently analyzed by FTIR and GC/MS. The FTIR spectrum indicated the presence of hydroxyl groups having a transmittance at 3337 cm^−1^ as well as the presence of a carboxyl group at 1696 cm^−1^. The transmittance at 961 cm^−1^ confirmed that the product was composed of trans-unsaturation (Figure 1). The peaks identified by FTIR corroborate with those obtained previously on DOD [26].

**Figure 2:**
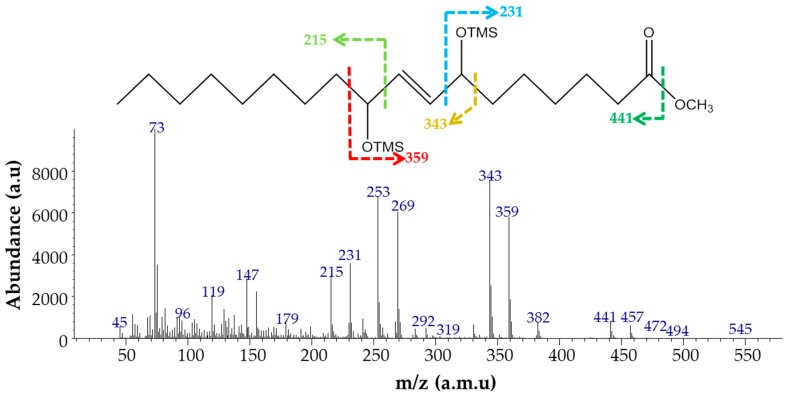
EI mass spectra of the major product produced from olive oil by *Pseudomonas aeruginosa* PR3.

The extract was methylated by diazomethane and derivatized using a mixture of TMSI and pyridine prior to GC/MS analysis. The GC profile showed one main peak with a retention time of 15.503 min, and the area percentage of this peak was 94% of the total peaks excluding the internal standard (data not shown). Figure 2 shows the electron impact MS analysis of methylated TMS derivatives. The peak at *m*/*z* 215 represents a fragment containing one TMS group, while the peak at *m*/*z* 343 represents a fragment containing two TMS groups and a double bond. Two other significant peaks at *m*/*z* 231 and *m*/*z* 359 represent two fragments involved in the methylated carboxyl group containing one TMS group and two TMS groups with a double bond, respectively. The presence of a peak at *m*/*z* 472 can be attributed to the molecular mass of the TMS derivative of the methylated product. The plausible mass fragmentation routes (colored arrows) are also graphically represented in Figure 2. These fragments assigned a double bond at C8-9 and two hydroxyl groups at C7 and C10. The fragmentation patterns obtained through GC/MS analysis support previous observations made for DOD identification [22,23]. The results obtained by FTIR and GC/MS confirm the bioconversion of olive oil to DOD by strain PR3.

In this study, olive oil was used as a model substrate and can be replaced by many other cheap vegetable oils containing high oleic acid content (e.g., sunflower oil, canola oil, and palm oil containing 82%, 61%, and 36.6% oleic acid, respectively). Recently, DOD production was reported using Philippine nut oil and palm oil, demonstrating that cheap oil substrates may also be used for this purpose [28].

### 3.2. Synthesis of PUs from DOD (PU-DOD)

**Figure 3:**
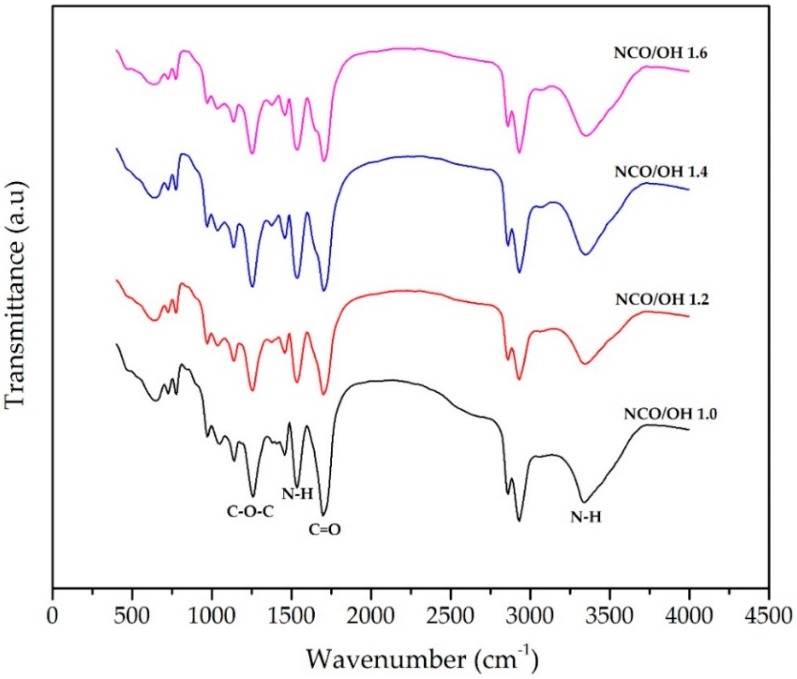
FTIR spectra of polyurethane-7,10-dihydroxy-8(E)-octadecenoic acid (PU-DOD) with different NCO/OH ratios.

Since the DOD produced through the bioconversion of olive oil contains two hydroxyl groups, the potential for PU formation using DOD was evaluated. To achieve this, PU-DOD was prepared by reacting DOD and HMDI with four different NCO/OH ratios (1.0, 1.2, 1.4, and 1.6). Preliminary experiments performed at low NCO/OH ratios of less than 1.0 showed that DOD could not be effectively linked with HMDI and that the sample remained in a liquid state. On the other hand, when the ratio exceeded 1.6, the polymer became more rigid and stuck to the glass plate. The FTIR spectrum of PU-DOD prepared from DOD and HMDI is shown in Figure 3.

The bands located at 3410 cm^−1^ and 1537 cm^−1^ represent the N–H group (N–H stretching and N-H bending, respectively). The amide carbonyl stretch at about 1699 cm^−1^ and the N–H bending vibration coupled to the C–N stretch (at 1130 cm^−1^) arise possibly due to the reaction of the carboxyl group and –N=C=O [29]. The presence of a band at 1254 cm^−1^ of the C–O–C group confirms the formation of PU because both DOD and HMDI do not contain C–O–C bonds. The absence of a band at 2270 cm^−1^ of the N=C=O group confirms that all HMDI reacted with OH groups during polymerization [30,31].

**Figure 4:**
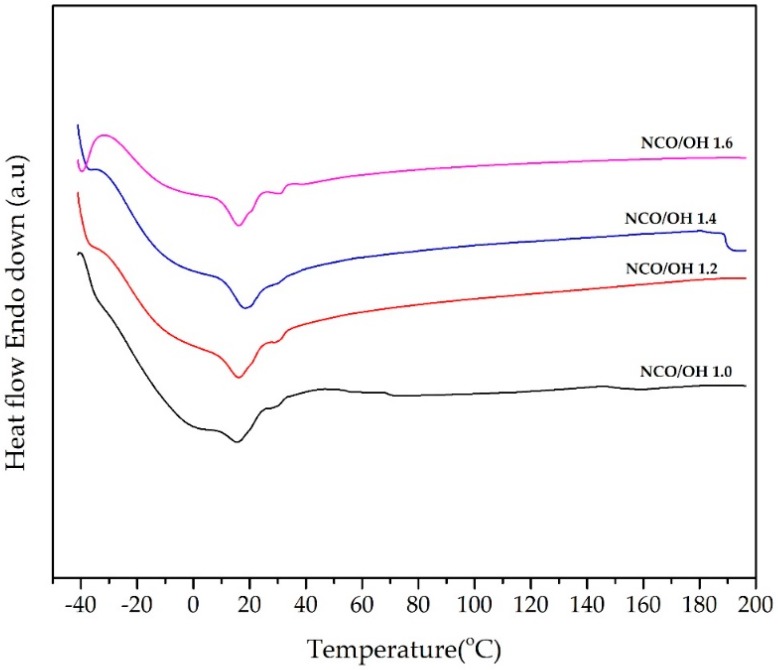
Differential scanning calorimetry (DSC) analysis of PU-DOD with different NCO/OH ratios.

The glass transition temperature (*T_g_*) of PU–DOD was also measured. *T_g_* was taken as the mid-point or steepest slope of the inflection with the onset and outset temperatures defining the *T_g_* range [10,12]. The data is shown in Figure 4 and summarized in Table 1. The *T_g_* of PU-DOD increased with increasing NCO/OH ratio to 1.4. The *T_g_* of the polymer was 11.3 °C at a ratio of 1.0 and reached 13.6 °C at a ratio of 1.4 (Table 1). As the ratio of NCO/OH increased, the reaction between the NCO and OH groups increased. There was no peak corresponding to the melting temperature.

**Figure 5:**
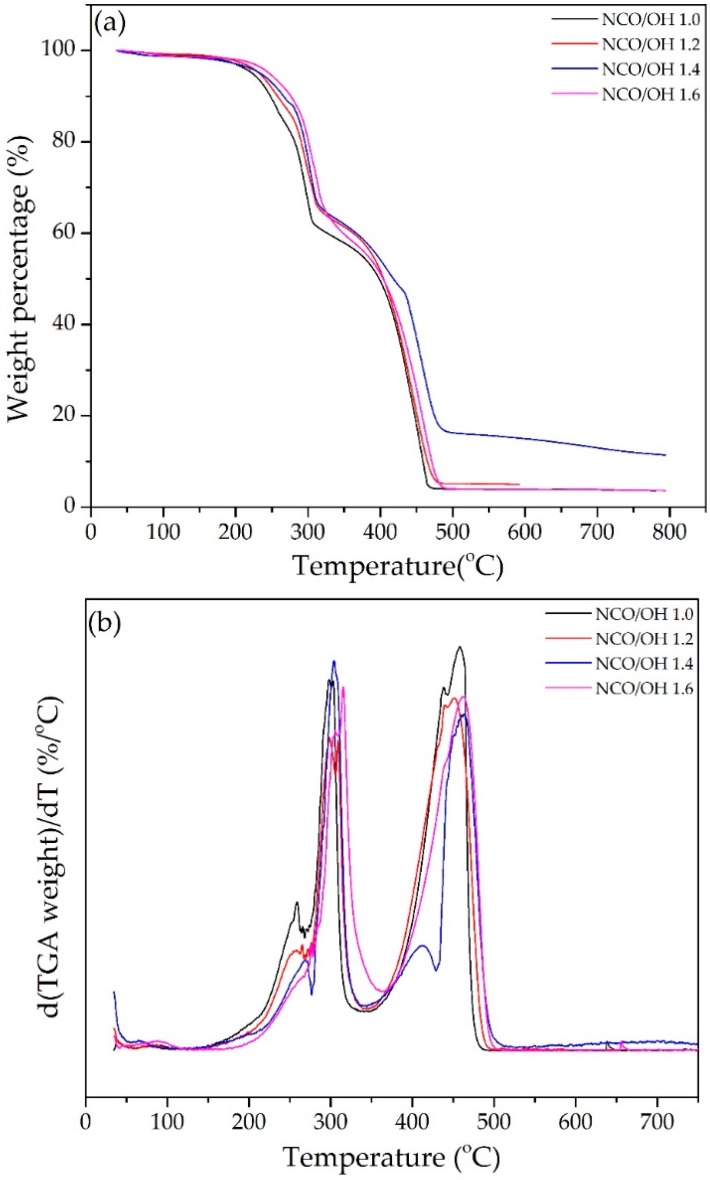
(**a**) Thermogravimetric analysis (TGA) curves and (**b**) their derivative curves of PU-DOD with different NCO/OH ratios.

The TGA and DTG weight loss curves of PU-DOD with different ratios of NCO/OH are shown in Figure 5. For all PU-DOD samples, decomposition began at 200 °C and took place via three steps. The first step occurred in the range of 200–320 °C, which is related to the decomposition of unstable urethane bonds, e.g., the separation of NCO and OH groups, and the formation of primary and secondary amines. The second step was detected in the range of 320–470 °C, which corresponds to the chain scission of polyols. The third step was started at a temperature higher than 470 °C (Figure 5). The existence of the third step at 470 °C can be explained by the decomposition of the fragments formed before the second step [32]. In addition, the 10% weight loss temperature (*T*_10%_) increased with increasing NCO/OH ratio (Table 1) due to the possibility of trimer formation of the diisocyanate compound [30]. The temperatures of the maximum DTG peaks (*T*_max_) for PU-DOD are slightly higher when the NCO/OH ratio is high.

**Figure 6:**
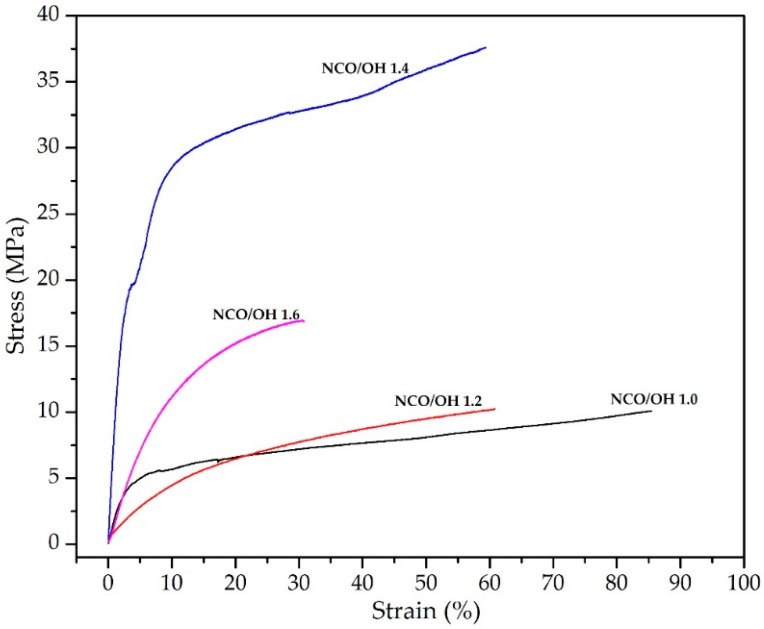
Stress-strain curves of PU-DOD with different NCO/OH ratios.

The tensile properties of the PU-DOD polymers obtained at different NCO/OH ratios are summarized in Table 1. As mentioned earlier, PU-DOD became more rigid with increasing NCO/OH ratio, which explains why the elongation at break decreased with increasing the ratio. The elongation at break decreased from 85.6% at a ratio of 1.0 to 31.2% at a ratio of 1.6. Apparently, the state of PU-DOD changed from ductile to brittle as the ratio increased. On the other hand, the tensile strength was the highest at 37.9 MPa when the NCO/OH ratio was 1.4 (Figure 6). Therefore, the optimum NCO/OH ratio can be considered to be important for PU synthesis. A further increase in the ratio to 1.6 lowered the tensile strength due to the formation of the trimer. As the NCO/OH ratio increased, the trimer content increased and the polymer became more brittle, which increased the tensile strength and decreased the elongation at break [30]. Such brittleness may also be attributed to the amide formation due to the reaction between the carboxyl group (of DOD) and NCO (of HMDI). Such a reaction may occur even at room temperature and involves mixed carbamic-carboxylic anhydride intermediates as described by others [29]. The carbon of CO_2_ generated during the reaction originates from isocyanate, although the exact mechanism is yet to be reported.

### 3.3. Synthesis of PUs from DOD and PEG or PCLDO (PU-DOD/PEG or PU-DOD/PCLDO)

**Figure 7:**
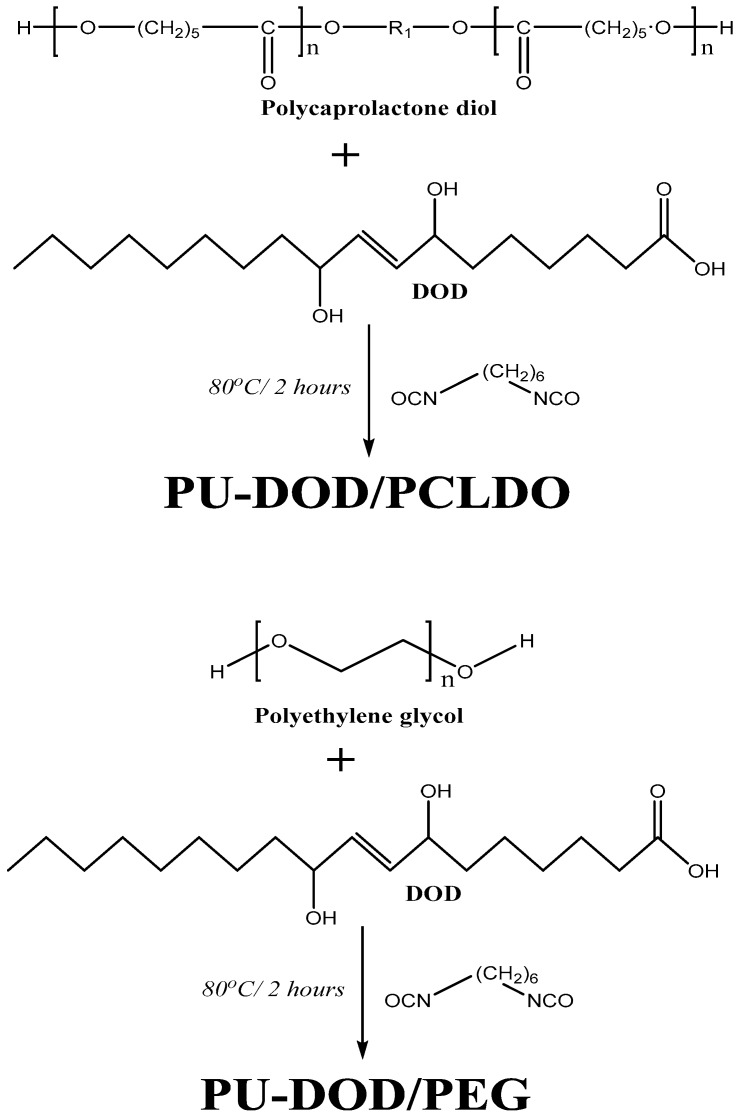
Reaction schemes for the synthesis of PU-DOD/ polycaprolactone diol (PCLDO) and PU-DOD/ polyethylene glycol (PEG).

Attempts were made to enhance the diversity of the PU-DOD polymers. To achieve this, three kinds of PEG (molecular weights 200, 2000, 20,000) or PCLDO (molecular weight 2000) were mixed with DOD and reacted with HMDI to form new PUs termed as PU-DOD/PEG200, PU-DOD/PEG2K, PU-DOD/PEG20K, and PU-DOD/PCLDO, respectively. The chemical structures of DOD, PEG, and PCLDO as well as the reaction schemes to form PUs are shown in Figure 7.

**Figure 8:**
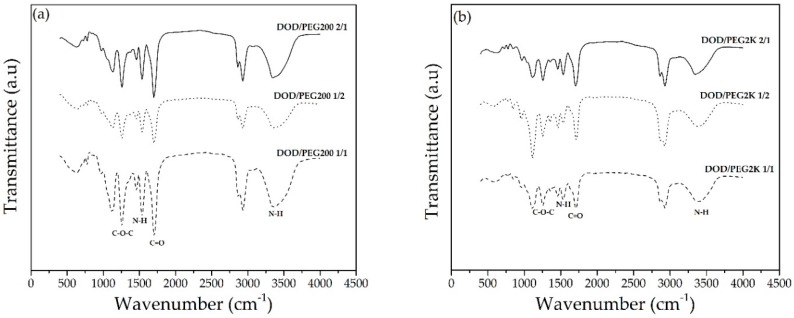

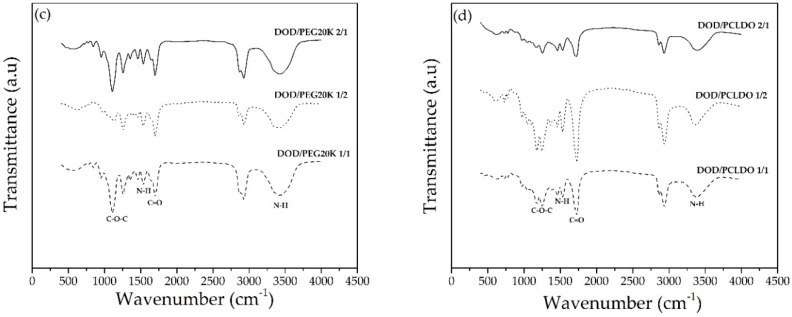
FTIR spectra of (**a**) PU-DOD/PEG200, (**b**) PU-DOD/PEG2K, (**c**) PU-DOD/PEG20K, and (**d**) PU-DOD/PCLDO.

The weight ratios of DOD to PEG or PCLDO were maintained at 2/1, 1/1, and 1/2. The FTIR of all of these samples indicated the formation of PUs by peaks at 3400 cm^−1^ and 1550 cm^−1^ of the N–H group, a peak around 1700 cm^−1^ of the C=O group, and a peak at 1250 cm^−1^ of the C–O–C group (Figure 8). There was no stretching vibration band at 2270 cm^−1^ indicating the absence of the N=C=O group [31]. All of these observations resemble typical FTIR spectra of PU.

**Figure 9:**
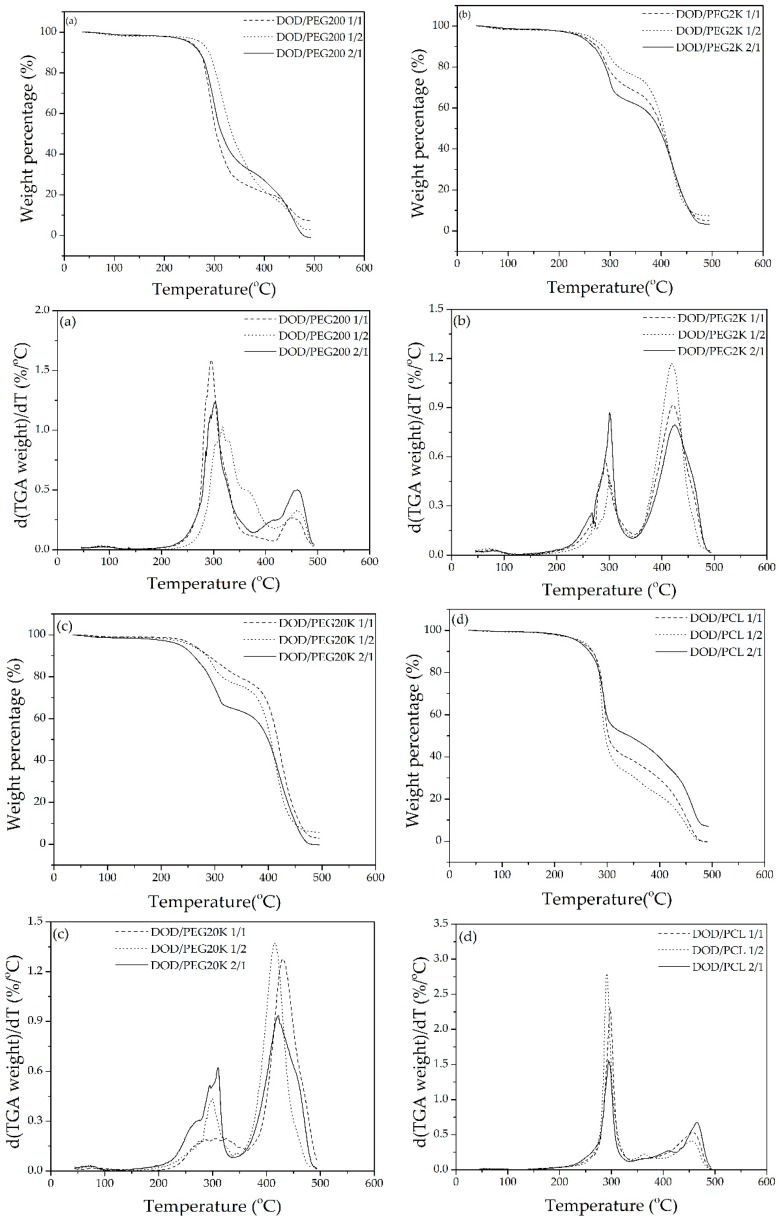
TGA curves and their derivative curves of (**a**) PU-DOD/PEG200, (**b**) PU-DOD/PEG2K, (**c**) PU-DOD/PEG20K, and (**d**) PU-DOD/PCLDO.

The TGA curves of all polymeric samples displayed a slow degradation at the beginning and then a rapid decomposition process (Figure 9). Their degradation included three steps similar to PU-DOD: the first step from 230 °C to 350 °C due to the decomposition of unstable urethane bonds, followed by the second step from 350 °C to 470 °C related with the degradation of the polyol segments, and finally the last step higher than 470 °C where the sample was completely decomposed.

Figure 9 compares various PUs possessing three hard segment contents based on the weight loss with temperature. It is evident that the first degradation step varies within 5–15 °C for PU-DOD/PCLDO and PU-DOD/PEG, indicating the role of the hard segment structure during the first step of degradation. In this context, other chain extenders such as 1,3-propanediol and 1,4-butanediol also show promising results and are potential candidates for bio-based PUs [31]. The *T*_10%_ of PU-DOD/PEG and PU-DOD/PCLDO increased with increasing the fraction of PEG or PCLDO in the polymer. Among these polymers, PU-DOD/PEG20K had the highest *T*_10%_ (290 °C) and PU-DOD/PCLDO had the lowest *T*_10%_ (273 °C). Thus, it can be concluded that PU-DOD/PEG20K possesses higher thermal stability than the others [33].

At a temperature higher than 160 °C, urethane bonds reach a simultaneous reversible state of dissociation and re-association, as reported previously [32]. Depending on the polymer type, this phenomenon turns irreversible at about 200 °C. Therefore, it can be assumed that during the first-stage urethane bond, degradation occurs and the urethane function with HMDI is less stable. Meanwhile, the second degradation step at a similar temperature shows the degradation of polyol, the soft polyurethane segment. A similar observation was previously made for vegetable oil-based PU [9,32]. Lastly, the third stage of degradation occurs at a similar temperature range of 415–465 °C, depending on the PU type. This could be related to the disintegration of fragments produced in the previous stage.

**Figure 10:**
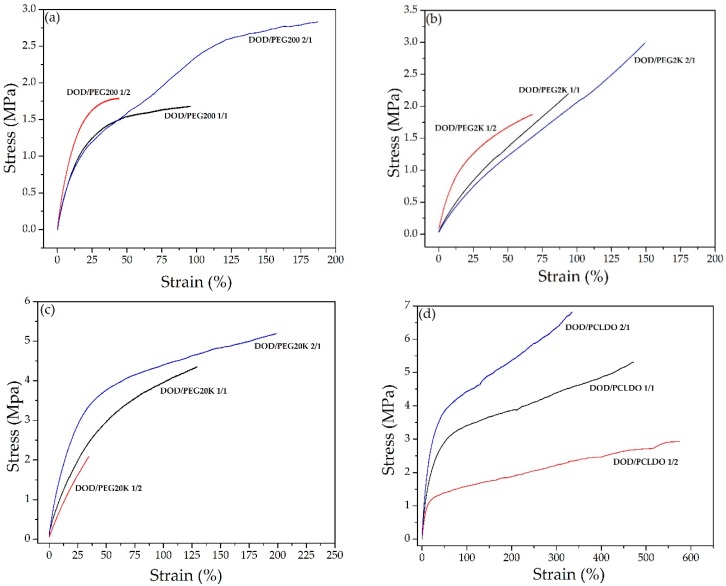
Stress-strain curves of (**a**) PU-DOD/PEG200, (**b**) PU-DOD/PEG2K, (**c**) PU-DOD/PEG20K, and (**d**) PU-DOD/PCLDO.

Figure 10 shows the stress-strain curves of PU films based on various combinations of DOD and PEG/PCLDO. When DOD was combined with PEG, the polymer displayed quite elastic regions in the beginning. In the curves of PU-DOD/PCLDO, elastic regions are initially observed, followed by yield points and plastic behavior. The increasing content of PCLDO within the polymer exhibited a longer plastic region.

The tensile strength and elongation at break of all PU-DOD/PEG and PU-DOD/PCLDO samples were also evaluated compared to PU-DOD at the same NCO/OH ratio of 1.4 (Table 2). The tensile strength of PU-DOD/PCLDO was lower while the elongation at break was higher compared to PU-DOD (Figure 10). PCLDO decreased the tensile strength by increasing the soft segment of PU when mixed with DOD. As the fraction of PCLDO increased, the elongation at break of PU-DOD/PCLDO increased from 335% to 576% and the tensile strength decreased from 6.83 MPa to 2.92 MPa. The elongation at break of PU-DOD/PEG ranged from 34% to 200% and the tensile strength increased with increasing molecular weight of PEG. The highest tensile strength of PU-DOD/PEG200 was 2.83 MPa and reached 5.18 MPa for PU-DOD/PEG20K. Mechanical properties data show that the hardness decreases as the content of other polyols increases during the synthesis of PUs from DOD and other polyols. In all cases, a polyol mixture of 2:1 displayed the highest tensile strength and elongation at break, except when PCLDO was used with DOD. PU-DOD/PCLDO showed the characteristics of elastomers with high elongation (over 335%) and low tensile strength (below 7 MPa), while PU-DOD/PEG showed plastic properties with a lower elongation at break (below 200%). The TGA results show that PU-DOD/PEG20K had higher thermal stability than the other polymers.

## 4. Conclusions

A bio-based polyol, DOD, was produced by *P. aeruginosa* PR3 using olive oil as a substrate. PUs prepared from DOD with an NCO/OH ratio of 1.4 were found to be amorphous polymers with an elongation at break and tensile strength of 59.3% and 37.9 MPa, respectively. Mixing DOD with PCLDO or PEG at different weight ratios further enhanced the diversity of PU-DOD. This study demonstrates that natural oils can be effectively bio-converted to HFAs, which can act as building blocks to synthesize bio-based PUs.

## Figures and Tables

**Table 1:** Thermal and mechanical properties of PU-DOD.

NCO/OH Ratio	*T_g_* (°C)	TGA in Nitrogen (°C)			Elongation at Break (%)	Tensile Strength (MPa)
		*T*_10%_	*T*_50%_	*T*_max_ (first/second)		
1.0	11.3	248	398	298/458	85.6 ± 3.77	10.1 ± 1.58
1.2	12.0	260	407	298/452	60.8 ± 1.12	10.3 ± 0.14
1.4	13.6	262	414	304/463	59.3 ± 4.01	37.9 ± 4.40
1.6	12.3	272	400	315/461	31.2 ± 1.12	16.9 ± 1.36

**Table 2:** Thermal and mechanical properties of PUs from DOD with PEG or PCLDO.

Weight Ratio of DOD to PEG or PCLDO	TGA in Nitrogen (°C)			Elongation at Break (%)	Tensile Strength (MPa)
	*T*_10%_	*T*_50%_	*T*_max_ (first/second)		
**PU-DOD/PEG200**					
2/1	266	313	303/461	187 ± 5.22	2.83 ± 0.19
1/1	272	304	296/449	95.4 ± 9.74	1.68 ± 0.12
1/2	285	336	318/460	45.5 ± 3.59	1.79 ± 0.23
**PU-DOD/PEG2K**					
2/1	270	395	301/425	149 ± 5.06	2.99 ± 0.15
1/1	276	401	293/421	94.0 ± 5.07	2.21 ± 0.28
1/2	287	405	299/419	68.9 ± 5.92	1.89 ± 0.38
**PU-DOD/PEG20K**					
2/1	273	401	310/422	200 ± 1.80	5.18 ± 0.82
1/1	285	417	299/425	129 ± 3.82	4.38 ± 0.33
1/2	290	409	300/415	34.7 ± 0.68	2.11 ± 0.18
**PU-DOD/PCLDO**					
2/1	266	342	295/465	335 ± 5.22	6.83 ± 0.24
1/1	272	302	297/457	472 ± 10.30	5.32 ± 0.68
1/2	273	296	291/453	576 ± 13.30	2.92 ± 0.41
